# Fine carrier frequency offset estimation for OFDM and MIMO-OFDM systems: A comparative study

**DOI:** 10.1038/s41598-025-98233-3

**Published:** 2025-05-05

**Authors:** Moatasem Mohammed Elsayed Kotb, Maha Raof Abdel-Haleem Mohamed, Ashraf Yahya Hassan Ali Fahmy, Ashraf Shawky Selim SayedAhmed Mohra

**Affiliations:** EE Dept. Benha faculty of engineering, Benha University, Benha, Egypt

**Keywords:** Multiple-Input and Multiple-Output (MIMO) technology, Orthogonal Frequency-Division multiplexing (OFDM), Frequency synchronization, Carrier Frequency Offset (CFO) Estimation, Data aided (DA) techniques, Machine learning for CFO Estimation, Kernel Support Vector Machine (KSVM), Linear Discriminant Analysis (LDA), Artificial Neural Network (ANN), Adaptive filter, Additive White Gaussian Noise (AWGN), Flat Rayleigh fading channel, Frequency-Selective Rayleigh fading channel, Supervised learning techniques, Engineering, Electrical and electronic engineering

## Abstract

In Orthogonal Frequency Division Multiplexing (OFDM) and Multiple-Input Multiple-Output-OFDM (MIMO-OFDM) systems, estimating Carrier Frequency Offset (CFO) is a critical challenge, particularly in degraded channel conditions where traditional methods struggle with precision and adaptability. This comparative study views various existing CFO estimation techniques and identifies three conventional methods—CFOest, CC, and AF—as benchmarks. To enhance estimation accuracy, a machine learning-based approach is proposed to effectively function across different channel conditions. Three distinct CFO estimators are developed using Kernel Support Vector Machine (KSVM), Linear Discriminant Analysis (LDA), and Artificial Neural Network (ANN), as this is a common strategy in machine learning for identifying optimal solutions. A comparative analysis of their performance demonstrates that the proposed approach outperforms traditional techniques by achieving lower Root Mean Square Error (RMSE), with the ANN-based CFO estimator performing best in larger estimation ranges, while the KSVM-based estimator excels in smaller ranges. To further enhance accuracy, a novel three-step machine learning-based approach is proposed, demonstrating significant improvements in accuracy through subsequent simulations when contrasted with conventional methods and single-step models.

## Introduction

Multiple-input multiple-output orthogonal frequency division multiplexing (MIMO-OFDM) and orthogonal frequency division multiplexing (OFDM) are key wireless communication technologies that enable high data rates and efficient spectrum utilization by combining MIMO’s spatial diversity with OFDM’s robust subcarrier-based modulation^1^. However, a major challenge in these systems is carrier frequency offset (CFO), which arises from mismatches between the received signal’s carrier frequency and the receiver’s local oscillator, often due to synchronization errors and Doppler shifts. CFO introduces inter-carrier interference (ICI) and inter-symbol interference (ISI), leading to transmission errors, reduced signal-to-noise ratio (SNR), and degraded overall performance^2^. Effective CFO compensation is essential for minimizing these distortions, ensuring accurate symbol timing, and optimizing channel estimation. Addressing CFO not only enhances system reliability but also maximizes data throughput and multi-user efficiency, making it a fundamental aspect of OFDM and MIMO-OFDM system design.

Various techniques can mitigate the impact of Carrier Frequency Offset (CFO), broadly classified into data-aided (DA) and non-data-aided (NDA) methods^3^. DA methods rely on known pilot symbols or predefined data structures to estimate and compensate for CFO with high accuracy, while NDA methods use statistical analysis and modulation properties without requiring specific data, often trading precision for lower complexity. The choice between these approaches depends on factors such as system requirements, data availability, computational complexity, and the balance between accuracy and implementation feasibility. One effective CFO removal technique is the use of adaptive filters, which not only mitigate CFO but also address multipath interference, noise, and distortion in OFDM and MIMO-OFDM systems. These filters continuously adjust their coefficients based on the received signal’s characteristics, enhancing synchronization, equalization, and channel estimation while adapting to dynamic channel conditions for optimized signal reception. CFO estimation using adaptive filters can involve phase tracking, where coefficients align with the signal’s phase; error signal analysis, which refines phase deviations through feedback mechanisms like phase-locked loops; or direct measurement of CFO by monitoring coefficient variations over time. By effectively minimizing CFO and improving overall signal quality, adaptive filters play a vital role in ensuring reliable data transmission in wireless communication systems.

Supervised learning is an essential machine learning technique that enables models to make predictions or decisions based on labeled training data. It involves training on input-output pairs, where inputs represent features and outputs correspond to target values or labels^4^. In wireless communication, supervised learning plays a critical role in various tasks. It enhances channel estimation^5^ by using received signals and known training data to estimate channel parameters, mitigating fading, interference, and distortions. Additionally, it improves signal detection by learning the mapping between received signals and transmitted symbols, thereby enhancing communication reliability^6^. Supervised learning also optimizes resource allocation by understanding the relationship between resource distribution and performance metrics, enabling efficient power control, subcarrier assignment, and antenna selection to maximize system capacity while minimizing interference. Furthermore, it aids in interference management by identifying patterns, predicting interference levels, and adjusting transmission strategies to enhance system performance. Similarly, supervised learning techniques can be applied to estimate CFO by leveraging patterns in received signal characteristics and their corresponding CFO values. By training on features such as phase shifts, frequency deviations, and signal distortions, these models can capture intricate relationships that degrade the accuracy of conventional estimation approaches. Supervised learning offers the flexibility to incorporate diverse signal properties, adapt to varying channel conditions, and account for non-linear distortions, making it a versatile tool for CFO estimation.

Our research had four main objectives. First, we aimed to analyze the impact of CFO on the performance of OFDM and MIMO-OFDM systems, particularly its effects on system throughput and error rates. Second, we examined existing CFO estimation methods in both OFDM and MIMO-OFDM systems, highlighting key approaches that have been traditionally used. Third, we explored machine learning techniques for CFO estimation, focusing on enhancing their performance while keeping computational costs low. Finally, we worked on developing innovative algorithms to improve the accuracy of CFO estimation, ensuring more reliable and efficient system operation.

**Key Contributions of This Work**:

1. **Key Advantages of Our (Single step) Machine Learning-Based CFO Estimation Approach**: We introduce machine learning-based CFO estimation methods (Single-Step-KSVM-based CFO estimator, Single-Step-LDA-based CFO estimator, and Single-Step-ANN-based CFO estimator), alongside conventional data-aided techniques (CFOest, CC, and AF). Key benefits include:

- **Superior Accuracy Than Conventional Data-Aided Techniques** – Our machine learning-based CFO estimators (Single-Step-KSVM, Single-Step-LDA, and Single-Step-ANN) outperform conventional data-aided techniques (CFOest, CC, and AF) by achieving lower RMSE, ensuring more precise estimation.
- **Robustness in Challenging Conditions** – It remains highly effective in low SNR and complex channel environments where conventional techniques degrade, delivering reliable signal processing.
- **Adaptability to Dynamic Environments** – It efficiently handles rapid channel variations without compromising performance, making it suitable for highly dynamic wireless systems.
- **Flexibility Across Wireless Systems** – Supports a broad range of signal conditions and modulation schemes, enhancing applicability across various communication scenarios.
- **Optimized Computational Efficiency Compared to Heavily Computational Cost Methods Like CNNs** – By balancing accuracy and processing cost, our model is more practical and scalable for real-world applications. Unlike deep learning methods such as CNNs, which require large datasets, high memory, and extensive processing power, our approach achieves high estimation accuracy with significantly lower computational overhead.
- **Feature Optimization for Enhanced Estimation** – Leveraging selected input features, the model captures both amplitude and phase distortions, preserving I/Q information, normalizing magnitude for better generalization, and stabilizing phase representation, leading to accurate CFO estimation.

2. **Enhanced Accuracy via a Novel Three-Step Classifier Framework**: We also propose a three-step classifier-based CFO estimation technique that builds on the strengths of single-step machine learning methods while achieving better accuracy. By leveraging a structured multi-step framework, our Three-Step approach improves estimation accuracy while preserving adaptability to diverse channel conditions and robustness in low SNR and degraded channel environments.
3. **Comprehensive Evaluation and Robustness to Channel Variations**: Extensive Monte Carlo simulations under AWGN, Flat Rayleigh fading, and Frequency-Selective Rayleigh fading confirm that our machine learning-based approaches achieve consistently lower Root Mean Square Error (RMSE) compared to conventional methods, showcasing their robustness against noise and channel impairments.
4. **Potential to Replace Existing CFO Estimation Techniques**: Simulation results confirm the superior accuracy and resilience of our methods in challenging channel conditions, highlighting their potential to replace existing CFO estimation techniques. However, in real-world deployments, the choice of estimator must balance accuracy, computational cost, and processing time based on the application’s specific requirements. While some estimators offer high accuracy, others achieve comparable performance with lower processing time, making the selection dependent on the specific needs of each application.

The structure of the paper is outlined as follows: Section II provides an overview of the prior research, Section III introduces the Proposed method, Section IV discusses the results and provides analysis, and finally, Section V presents the concluding remarks.

## Survey

Previous studies on carrier frequency offset (CFO) estimation have explored various techniques, each employing different approaches to improve accuracy and efficiency. Moose^7^ introduced a method using the cyclic prefix, where CFO was estimated by comparing the phase of the received signal to its expected phase, followed by correction through adjustments to the receiver’s local oscillator. Schmidl and Cox^8^ proposed an approach that inserted a known pilot symbol sequence into the transmitted OFDM signal, allowing the receiver to estimate CFO by correlating the received signal with the pilot sequence. Mody and Stuber^9^ developed a multi-step synchronization process that included coarse time synchronization to determine the start of the OFDM frame, followed by frequency offset estimation and fine synchronization using two identical back-to-back OFDM symbols.

Luise et al.^10^ presented a low-complexity multi-stage method for CFO recovery, beginning with timing synchronization to identify the start of each OFDM symbol, followed by coarse frequency estimation using the cyclic prefix, fine frequency estimation through a two-step correlation algorithm, and frequency tracking via phase accumulation. Lei and Ng^11^ estimated CFO by leveraging the correlation between received pilot tones, utilizing known pilot tone spacing and expected correlation properties to achieve a more consistent estimation. Zhang et al.^12^ exploited the Hermitian symmetry property of OFDM signals to estimate CFO efficiently, transforming the received signal into the frequency domain using a fast Fourier transform and iteratively refining phase adjustments based on an optimization criterion.

Yu and Su^13^ formulated CFO estimation as an optimization problem, comparing received pilot symbols with their expected values and maximizing the likelihood function to align them accurately. Shi and Serpedin^14^ introduced a fast, low-complexity frame and carrier acquisition scheme using a maximum likelihood-based correlation metric to efficiently detect the CFO. Yao and Giannakis^15^ proposed a blind CFO estimation technique applicable to single-input single-output (SISO), multiple-input multiple-output (MIMO), and multiuser OFDM systems, leveraging the OFDM signal structure to estimate CFO without explicit pilot symbols. Yi et al.^16^ used a maximum likelihood estimator for frequency synchronization, incorporating minimum energy detection for coarse CFO estimation and partial correlation for fine estimation.

Fan et al.^17^ employed two identical weighted sequences to estimate coarse fractional CFO, followed by redundant cyclic prefixes for fine fractional CFO estimation. Marrero et al.^18^ introduced a blind adaptive filter approach that combined equalization and constellation symbol recovery for phase shift keying (PSK) waveforms, offering reduced computational complexity and faster convergence. Liu and Wang^19^ focused on CFO estimation in wireless sensor networks, integrating multitask learning (MTL) with channel residual energy (CRE) to minimize power consumption and inference time while mitigating the coupling effect of CFO and IQ imbalance. Cheng et al.^20^ developed an adaptive algorithm combining set-membership filtering with real-time adjustments to counteract channel impairments, IQ imbalance, and CFO, particularly in short cyclic prefix scenarios.

These studies demonstrate the diverse range of CFO estimation techniques, from pilot-based and blind estimation methods to optimization-driven and machine learning-based approaches, each addressing different challenges in CFO estimation across various communication systems. Table 1 summarizes the previous work on CFO removal.

**Table 1:** Survey summary.

Paper / Authors	Problem	Technique	Drawbacks
Moose, P. H.^7^	Frequency offset correction	This method uses the cyclic prefix in OFDM systems to estimate and correct frequency offset. By comparing the phase of the cyclic prefix and its corresponding data symbols, it computes the frequency offset, which is then used to adjust the local oscillator. This technique is simple and effective for small frequency offsets.	Degraded performance in low SNR and under more difficult channel conditions.
Schmidl, T. M., and D. C. Cox^8^	Frequency & timing synchronization in OFDM systems	This method employs a known pilot sequence embedded in the OFDM symbol to perform frequency and timing synchronization. The correlation of the received signal with the known pilot sequence allows the estimation of frequency offset and synchronization parameters, making it robust in many practical scenarios	More susceptible to significant frequency offsets, exhibiting reduced performance in low signal-to-noise ratios and under more difficult channel conditions.
Mody, A. N., and G. L. Stuber^9^	Synchronization for MIMO OFDM Systems	This method involves a sequential approach to synchronization in MIMO-OFDM systems. It begins with coarse time synchronization, followed by frequency offset estimation, and concludes with fine synchronization. Each stage progressively refines the synchronization accuracy.	–
Luise, M., et al.^10^	Low-complexity blind carrier frequency recovery	A low-complexity blind carrier frequency recovery method that does not rely on pilot signals. It consists of a multi-stage process, including timing synchronization, coarse frequency offset estimation, fine CFO estimation, and frequency tracking, designed to handle frequency misalignment in OFDM systems.	Decreased performance in highly dynamic environments or in the presence of significant channel variations.
Jing Lei and Tung-Sang Ng^11^	OFDM carrier frequency offset estimation	This method estimates carrier frequency offset by analyzing the correlation between pilot tones at known spacing in the frequency domain. By exploiting the periodicity of pilots, it accurately computes the offset.	–
Zhang, Z., et al.^12^	CFO estimation algorithm in OFDM systems	This algorithm uses Hermitian symmetry in the frequency domain for CFO estimation. Iterative optimization enhances estimation accuracy, making it computationally efficient while maintaining robustness against noise.	–
Yu, J. H., and Y. T. Su^13^	Frequency-offset estimation for OFDM systems	A pilot-assisted method that uses a maximum-likelihood estimation framework. Pilots embedded in the OFDM symbol are used to estimate frequency offset, offering high accuracy at the cost of spectral efficiency.	–
Shi, K., and E. Serpedin^14^	Coarse frame and carrier synchronization	This method uses correlation metrics to identify the peak for synchronization. It leverages maximum likelihood principles to estimate coarse frame timing and carrier synchronization, ensuring robustness against noise.	–
Yao, Y., and G. B. Giannakis^15^	Blind CFO estimation in SISO, MIMO, and multiuser OFDM	A blind estimation technique that relies on the inherent structure of the OFDM signal rather than explicit pilot symbols. It is designed for systems with no dedicated pilot tones, using signal properties for CFO estimation.	Blind estimation techniques, while pilot-free, are less accurate and may struggle with convergence in scenarios with severe multipath fading or strong noise interference.
Yi, Guo, et al.^16^	Time and frequency synchronization	This approach employs fractional CFO estimation using maximum likelihood methods combined with partial correlation techniques. It refines synchronization accuracy through fine estimation.	–
Fan, Xinge, et al.^17^	Time-frequency synchronization in OFDM	CAZAC (Constant Amplitude Zero Auto-Correlation) sequences are used to enhance timing and frequency synchronization. These sequences provide high correlation accuracy, especially under low SNR conditions, improving synchronization performance.	–
Marrero, Liset Martínez, et al.^18^	Single-step blind adaptive filter solution for constellation symbol recovery	This method uses a blind adaptive filter for constellation symbol recovery. It exploits the constant amplitude property of PSK signals and employs a novel coefficient update mechanism to recover symbols without requiring pilot signals.	The single-step blind adaptive filter is limited by its dependence on the constant amplitude property of PSK signals, making it unsuitable for higher-order modulations like QAM.
Liu, Siqi, and Shaowei Wang^19^	Carrier Frequency Offset Estimation	This method integrates MTL with CRE analysis to estimate CFO. The combination of tasks enables cost-effective and efficient frequency offset estimation.	–
Cheng, Nan-Hung, et al.^20^	Adaptive CFO estimation in interference environments	Adaptive CFO estimation is achieved using a set-membership filtering algorithm. It dynamically adjusts to interference and short cyclic prefix conditions in real-time, offering robust performance in varying environments.	–

Based on the reviewed literature, we can conclude that the main methods employed for carrier frequency offset estimation fall into four main categories: pilot-based CFO estimation, correlation-based methods, adaptive filtering techniques, and machine learning-based approaches.

## Proposed method

**Fig. 1:**
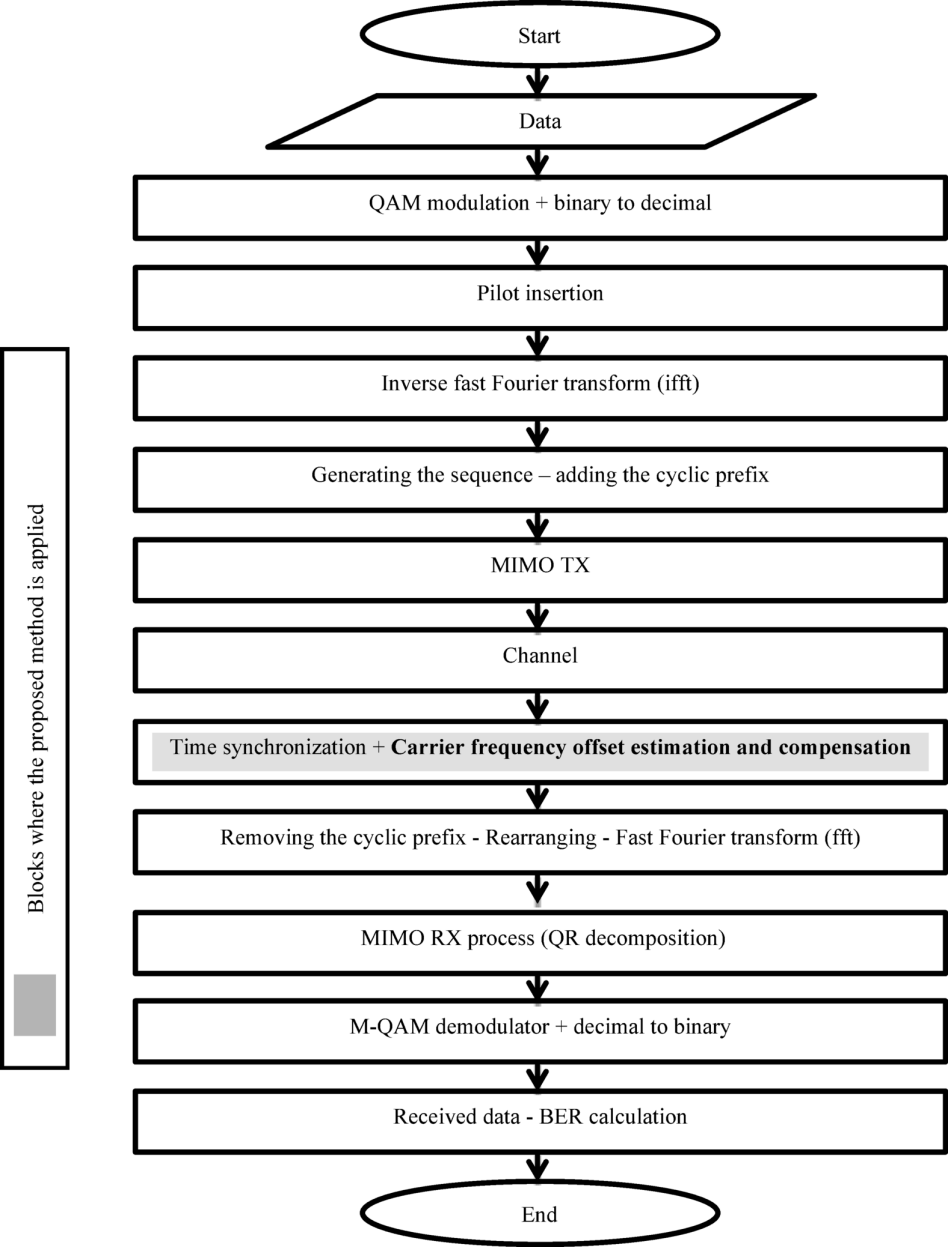
System block diagram.

Figure 1 illustrates the system’s block diagram. Certain blocks within the diagram can be easily modified by loosening them. This modification enables the transformation of MIMO-OFDM blocks into an OFDM system.

The proposed method section consists of two subsections. Subsection A focuses on the influence of the CFO on the system performance, while subsection B outlines the methods employed to eliminate this influence.

A. ***The impact of CFO on the system***.

1. ***OFDM system***.

In a one-input OFDM system, the signal received on the k-th subcarrier can be expressed as:

1 $$\:{Y}_{k}={H}_{k}{X}_{k}{e}^{j2\pi\:{\Delta}\text{f}\:{T}_{s}k}\:+\:\sum_{l\ne\:k}{H}_{l}{X}_{l}sinc\left(\pi\:{\Delta}\text{f}{\:T}_{s}\right(k-l\left)\right)+{N}_{k}$$

Where $\:{X}_{k}$ is the transmitted signal, $\:{H}_{k}$ represents the channel frequency response on subcarrier k, Δf is the carrier frequency offset, Ts is the OFDM symbol duration, and $\:{N}_{k}$ stands for additive noise.

The phase shift introduced by CFO, denoted as $\:{e}^{j2\pi\:{\Delta}\text{f}\:{T}_{s}k}$, causes rotation in the constellation points on the k-th subcarrier. Additionally, the term $\:sinc\left(\pi\:{\Delta}\text{f}{\:T}_{s}\right(k-l\left)\right)$ denotes inter-carrier interference arising from CFO, leading to interference between disparate subcarriers.

In OFDM system, CFO uniformly impacts all subcarriers, resulting in a consistent phase shift across them. This cumulative phase shift can lead to ICI between subcarriers over time, potentially causing errors if not properly rectified.

2) **MIMO-OFDM System**.

In MIMO-OFDM systems, each antenna pair may experience a different CFO, leading to inter-antenna interference (IAI) and inter-antenna coherence loss (IACM). To enhance system performance, accurate CFO estimation and compensation techniques are imperative to be performed on each antenna pair.

In a MIMO-OFDM system with perfect synchronization, the signal received by the r-th receiver on the k-th subcarrier, denoted as $\:{Y}_{r,k\:}$, can be expressed as:

2 $$\:{Y}_{r,k}=\sum_{t=1}^{{N}_{t}}{H}_{rt,k}{X}_{t,k}+{N}_{r,k}$$

Where $\:{N}_{t}$​ is the number of transmit antennas, $\:{H}_{rt,k}$ represents the channel frequency response from the t-th transmitter to the r-th receiver on the k-th subcarrier, $\:{X}_{t,k}$ denotes the transmitted signal from the t-th transmitter on the k-th subcarrier, and $\:{N}_{r,k}$ indicates the noise on the r-th receiver for the k-th subcarrier.

In the presence of a carrier frequency offset Δ between the transmitter and receiver oscillators, a phase shift occurs across every subcarrier. This phase shift adds up over the duration of the OFDM symbol, leading to ICI among the subcarriers. The signal received with a frequency offset of Δf can be expressed as:

3 $$\:{Y}_{r,k}=\sum_{t=1}^{{N}_{t}}{H}_{rt,k}{X}_{t,k}{e}^{j2\pi\:{\Delta}\text{f}\:{T}_{s}k}+\sum_{l\ne\:k}\sum_{t=1}^{{N}_{t}}{H}_{rt,l}{X}_{t,l}sinc\left(\pi\:{\Delta}\text{f}{\:T}_{s}\right(k-l\left)\right)+{N}_{r,k}$$

Where $\:{X}_{t,k}$ is the transmitted signal from the t-th transmit antenna on subcarrier k, $\:{H}_{rt,k}$ is the channel response from the t-th transmit antenna to the r-th receive antenna on subcarrier k, the exponential term $\:{e}^{j2\pi\:{\Delta}\text{f}\:{T}_{s}k}$ represents the CFO-induced phase shift in each transmit-receive path separately, $\:{N}_{r,k}$ is the noise on the r-th receive antenna for subcarrier k, *T*s represents the duration of an OFDM symbol, and the term $\:sinc\left(\pi\:{\Delta}\text{f}{\:T}_{s}\right(k-l\left)\right)$ describes the Inter-Carrier Interference arising from subcarriers $\:l\ne\:k$, indicating the influence of the Carrier Frequency Offset. Here, The ICI term includes contributions from all transmit antennas, leading to more complex ICI than in a single-input OFDM system.

The carrier frequency offset disturbs the orthogonality of the subcarriers and alters the effective channel matrix. With CFO taken into account, the effective channel matrix denoted as $\:{\widehat{H}}_{rt,k}$ for each subcarrier k can be roughly estimated as:

4 $$\:{\widehat{H}}_{rt,k}={H}_{rt,k}{e}^{j2\pi\:{\Delta}\text{f}\:{T}_{s}k}$$

The rotation in phase resulting from CFO variation alters the signal constellation upon reception, posing challenges to accurate decoding. With higher values of Δ*f*, the subcarriers’ orthogonality is more severely impacted, leading to a rise in error rates.

The power of ICI caused by the CFO in a MIMO-OFDM system is typically estimated as:

5 $$\:{P}_{ICI}\approx\:{P}_{signal}\left(1-{e}^{-{\left(\frac{2\pi\:{\Delta}\text{f}{T}_{s}}{N}\right)}^{2}}\right)$$

Where N denotes the quantity of subcarriers, while $\:{P}_{signal}$ represents the power of the transmitted signal.

The following figure illustrates the simulation of how CFO affects the performance of a MIMO-OFDM system.

**Fig. 2:**
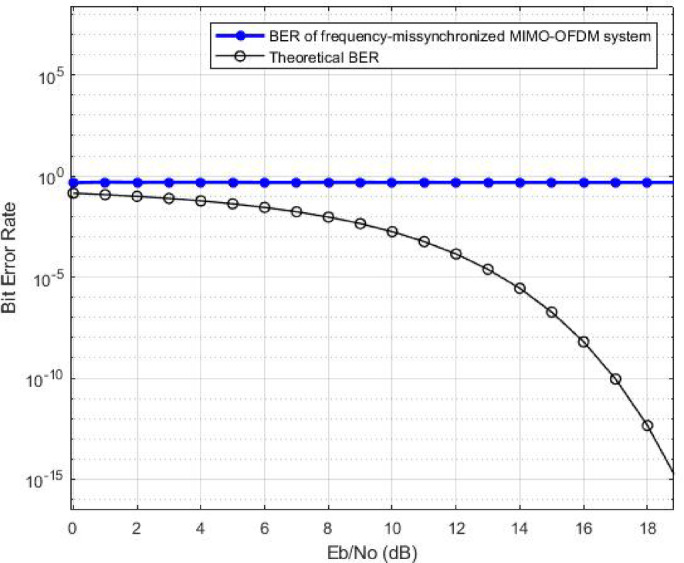
Impact of Carrier Frequency Offset on BER of a 16QAM-MIMO-OFDM system.

In this figure (Fig. 2), the “theoretical BER” represents the theoretical-synchronized BER of a 16QAM-MIMO-OFDM system, whereas the “BER of frequency misssynchronized MIMO-OFDM system” depicts the simulated system BER when affected by CFO = 1/500 (frequency offset normalized by the subcarrier spacing).

***B. The methods employed to eliminate the CFO***.

This subsection is organized into five parts: Subsections (1), (2), and (3) introduce the three-benchmark carrier frequency offset estimation methods, while Subsections (4) and (5) present Proposed method (1) and Proposed method (2) respectively.

1. **Frequency offset estimation (CFOet) technique**.

The observed OFDM symbols (preamble) in the time domain can be expressed as:

6 $$\begin{aligned}&{y}_{k}={X}_{k}{H}_{k}{e}^{j2\pi\:\varDelta fk{T}_{s}}+{N}_{k}\\&{z}_{k}={X}_{k}{H}_{k}{e}^{j2\pi\:\varDelta f(k+{N}_{c}){T}_{s}}+{N}_{k}\end{aligned}$$

Where $\:{X}_{k}$​ is the transmitted signal on subcarrier k, $\:{H}_{k}$​ is the channel frequency response for subcarrier k, $\:\varDelta f$ is the carrier frequency offset, $\:{T}_{s}$​ is the OFDM symbol duration, $\:{N}_{k}$​ represents additive noise, and $\:{N}_{c}$ is the number of subcarriers. The steps for applying the CFOest technique are as follows:

1. The ratio $\:{R}_{k}$​ between the consecutive two OFDM symbols is first calculated as:

7 $$\:{R}_{k}=\frac{{z}_{k}}{{y}_{k}}=\frac{{X}_{k}{H}_{k}{e}^{j2\pi\:\varDelta f(k+{N}_{c}){T}_{s}}+{N}_{k}}{{X}_{k}{H}_{k}{e}^{j2\pi\:\varDelta fk{T}_{s}}+{N}_{k}}$$

Neglecting noise ($\:{N}_{k}$​) for simplicity:

8 $$\:{R}_{k}\approx\:{e}^{j2\pi\:\varDelta f{N}_{c}{T}_{s}}$$

Then, the Averaging $\:{R}_{k}$ across all subcarriers is computed as:

9 $$\:\stackrel{-}{R}=\frac{1}{{N}_{c}-1}\sum_{k=0}^{{N}_{c}-2}{R}_{k}\approx\:{e}^{j2\pi\:\varDelta f{N}_{c}{T}_{s}}$$

2. The CFO is estimated as:

10 $$\:\varDelta f=\frac{\angle\:\left(\stackrel{-}{R}\right)}{2\pi\:{N}_{c}{T}_{s}}$$

2) **Cross-Correlation (CC) technique**.

This technique is widely recognized for estimating the CFO. The estimation process relies on correlating the received pilots with the target ones. The maximum correlation peak is then employed to estimate the CFO.

This can be represented as:

1. Cross-correlation:

11 $$\:{\text{r}}_{corr}=\sum_{n=0}^{N-1}{\text{x}}_{received}\left(n\right).{{p}_{target}\left(n\right)}^{*}$$

where $\:{\text{x}}_{received}$ is the received signal and $\:{p}_{target}$ is the target pilot sequence, and ∗ denotes the complex conjugate.

2. Peak index:

12 $$\:{k}_{peak}=\text{arg}\underset{k}{\text{max}}\left|{\text{r}}_{corr}\left(k\right)\right|$$

where $\:{\text{r}}_{corr}\left(k\right)$ represents the cross-correlation values across all time lags, and $\:{k}_{peak}$ ​is the index at which the correlation peak occurs.

3. CFO estimation:

13 $$\:\widehat{\theta\:}=\text{a}\text{r}\text{g}\left({\text{r}}_{corr}\right({k}_{peak}\left)\right)$$

where a $\:\text{a}\text{r}\text{g}\left({\text{r}}_{corr}\right({k}_{peak}\left)\right)$ gives the phase (argument) of the cross-correlation value at the peak index.

4. Normalized CFO:

14 $$\:{\widehat{f}}_{CFO}=\frac{\widehat{\theta\:}}{2\pi\:N}$$

where $\:N$ is the length of the pilots.

3) **Adaptive filtering (AF) method with phase difference analysis**.

The AF method with phase difference analysis is an effective approach for CFO estimation, leveraging the relationship between received and target pilot signals to refine CFO estimation. It is formulated as follows: Let x represent the column vector of received pilots with length N, and d represent the column vector of target pilots:

15 $$x=\left[\begin{array}{c}{x}_{1}\\\:{x}_{2}\\\:\begin{array}{c}\vdots\\\:{x}_{N}\end{array}\end{array}\right],\quad d=\left[\begin{array}{c}{d}_{1}\\\:{d}_{2}\\\:\begin{array}{c}\vdots\\\:{d}_{N}\end{array}\end{array}\right]$$

The filter weights (w) and the error signal (e) are both initialized as zero vectors:

16 $$\:w=\left[\begin{array}{c}0\\\:0\\\:\begin{array}{c}\vdots \\\:0\end{array}\end{array}\right],\quad e=\left[\begin{array}{c}0\\\:0\\\:\begin{array}{c}\vdots \\\:0\end{array}\end{array}\right]$$

For each *n* = 1,2,…,N, the output of the adaptive filter is computed as:

17 $$\:{y}_{n}={w}^{T}x$$

The error signal $\:{e}_{n}$​ is then calculated as the difference between the target pilot $\:{d}_{n}$​ and the filter output $\:{y}_{n}$:

18 $$\:{e}_{n}={d}_{n}-{y}_{n}$$

The filter weights are updated iteratively using the LMS algorithm:

19 $$\:{w}_{n+1}={w}_{n}+\:\mu\:x{e}_{n}^{*}$$

where $\:{e}_{n}^{*}$​ is the complex conjugate of the error signal $\:{e}_{n}$​ and µ is the learning rate of the adaptive filter.

The CFO can be estimated by computing the phase difference between the received pilots x and the error signal e. The CFO estimation is given by:

20 $$\:\stackrel{\sim}{{\upomega\:}}=\:-\frac{\text{a}\text{r}\text{g}\left(\sum_{n=1}^{N}{x}_{n}^{*}\:{e}_{n}\right)}{2\:{\uppi\:}\:\text{N}}$$

where $\:{x}_{n}^{*}$​ is the complex conjugate of $\:x\left(n\right)$, and arg(⋅) represents the phase angle of the sum.

4) **Proposed method (1) Single-Step-classifier-based CFO estimator**.

In our first approach (Method 1), we employed a classifier-based framework for CFO estimation, which, unlike traditional methods relying on closed-form solutions or iterative algorithms, leverages optimized feature mappings to effectively capture nonlinear relationships, reducing estimation errors and enhancing robustness. To enhance the accuracy and reliability of the model, we utilized multiple classifiers, a strategy commonly adopted in machine learning to identify the most effective solution. Additionally, we prioritized achieving optimal performance while keeping the computational cost as low as possible. As a result, computationally intensive models such as Convolutional Neural Networks (CNNs) were not considered, ensuring a balance between efficiency and effectiveness in our implementation. This led us to develop three different CFO estimators, each named after the classifier utilized: single step KSVM-based CFO estimator, single step LDA-based CFO estimator, and single step ANN-based CFO estimator. First the classifiers are introduced as follows:

***a) Kernel Support Vector Machine (KSVM)***.

A Kernel Support Vector Machine builds upon the linear SVM by utilizing kernel functions to process non-linear data through mapping it to a higher-dimensional feature space. This enhancement allows the algorithm to identify a hyperplane that optimizes the class margin, even when dealing with non-linearly separable data. The decision rule for an SVM can be expressed as^21^:

21 $$\:f\left(x\right)=sign\left(\sum_{i=1}^{N}{\alpha}_{i}{y}_{i}K\left({x}_{i},x\right)+b\right)$$

where $\:{\alpha}_{i}$​ are the Lagrange multipliers, $\:{y}_{i}\:$are the class labels, $\:{x}_{i}\:$are the support vectors, b is the bias term, and $\:K\left({x}_{i},x\right)\:$is the kernel function.

The Radial Basis Function (RBF) kernel, utilized in our approach, is defined as^21^:

22 $$\:K\left({x}_{i},x\right)=exp\left(-\gamma\:{||{x}_{i}-x||}^{2}\right)$$

Here, $\:\gamma\:\:>0$ is a kernel parameter that controls the influence of a single training example. Larger $\:\gamma\:$ values lead to more localized decision boundaries, while smaller values create smoother boundaries. The RBF kernel allows SVMs to represent intricate, non-linear decision boundaries by transforming data into a higher-dimensional space, making linear separation possible.

In our work, we utilize the Kernel Support Vector Machine to predict the CFO from the incoming signal. The Radial Basis Function kernel is employed to create nonlinear boundaries for accurate representation of the intricate connections between features and their classes. The KSVM assigns the selected features, defined at the end of Subsection C, to specific classes, each corresponding to a range of CFO values stored in a table. Training is performed over numerous frames to account for changing channel conditions and ensure optimal functionality. Following classification, a predefined table is used to convert the identified outcomes into the corresponding CFO values, finalizing the estimation procedure.

***b) Linear Discriminant Analysis (LDA)***.

Conducting LDA classification entails developing a discriminant analysis model to make predictions based on factors such as posterior probability, prior probability, and cost. The goal is to classify observations effectively by minimizing the expected classification cost. This minimization can be achieved by^22^:

23 $$\:\widehat{y}={\text{arg}min}_{y=1,\dots\:.,K}\:\sum_{k=1}^{K}\widehat{P}\left(k|x\right)C\left(y|k\right)$$

Where K is the number of classes, $\:\widehat{y}$ is the predicted classification, $\:\widehat{P}\left(k|x\right)$ is the posterior probability of class k for observation,$\:\:C\left(y|k\right)$ is the cost of classifying an observation to be y when its true class is k.

In our work, we utilize Linear Discriminant Analysis to predict the CFO of the incoming signals. The CFO estimation process involves LDA categorizing selected features, defined at the end of Subsection C, into predefined classes that represent different CFO values. To ensure stable numerical calculations and reliable classification results, we utilize the pseudo-inverse of the covariance matrix to handle situations where the matrices are ill-conditioned. The training phase occurs across multiple frames, adapting to varying channel conditions in order to optimize performance in different environments. After the classification phase, the outcomes are passed through a predetermined table that converts the results back to the corresponding CFO values, thereby finalizing the estimation process.

***c) Artificial Neural network (ANN)***.

The Multilayer Perceptron (MLP) is utilized for CFO prediction based on incoming signals. Comprising of the input, hidden, and output layers, MLP aims to classify incoming signals using the Backpropagation algorithm. In forward propagation, the input signal is processed through the network to generate an output, while backward propagation involves error calculation and adjusting weights accordingly. Activation functions and weights play crucial roles in mapping input signals to output in MLP, with the training process involving repeated forward and backward propagation to optimize weights and reduce errors.

The output of the j^th^ neuron in the (k + 1) layer is expressed as^23^:

24 $$\:{x}_{j}^{(k+1)}=R\left(\sum_{i=0}^{Mk}{W}_{ij}^{\left(k+1\right)}{x}_{i}^{k}\right)$$

Here, the parameters include R as the activation function, Mk as the count of nodes within the K^th^ layer, W as the weight connections among neurons, and $\:{x}_{i}^{k}$ as the output of the i^th^ neuron in the K^th^ layer.

The error function can be represented by the following expression^23^:

25 $$\:E=\frac{1}{2}\sum_{j=0}^{Mk}{({x}_{j}^{k}-{d}_{j}^{k})}^{2}$$

The intended outcome of the j^th^ neuron in the k^th^ layer is represented by $\:{d}_{j}^{k}$, while $\:{x}_{j}^{k}$ denotes the output of that same neuron in the k^th^ layer. In the training process, the Bayesian regularization back-propagation algorithm is employed, particularly utilizing the Levenberg-Marquardt optimization method^24^ to adjust weight and bias values. The ultimate goal is to enhance weights and reduce output error. The Levenberg-Marquardt optimization technique is widely recognized in numerical optimization, as it merges the strengths of the Gauss-Newton and gradient descent methods. This integration makes the Levenberg-Marquardt method a powerful means of minimizing nonlinear objective functions.

In our work, we utilize the MLP neural network to predict the CFO from the incoming signal. The MLP is designed with multiple hidden layers to model the complex, nonlinear relationships between the signal features and their corresponding different CFO values. During training, the network learns to map the input signal to the desired targets by adjusting its weights through an iterative optimization process. The training is performed over a large number of epochs to ensure the model adapts effectively to the underlying patterns in the data. Once trained, the MLP is used to classify or predict the CFO based on the learned representations, finalizing the estimation process.

**The training set for the classifier-based estimators is prepared as follows**:

Let N represent the number of pilots, and let $\:{\tau}_{true}$​ denote the true Carrier Frequency Offset. The input signal is a vector of length N, where each entry corresponds to the pilot value $\:\text{x}\left[\text{n}\right]$.

26 $$\:\text{x}\left[\text{n}\right]=\left(a+jb\right)\:\:\:\:\:\:for\:\:n=\text{0,1},2,\dots\:,N-1$$

To generate the training set, $\:{\tau}_{true}$​ is varied over a predefined range of values. The pilots affected by the CFO are given by:

27 $$\:{y}_{\tau\:}\left[n\right]={e}^{-i2\pi\:n{\tau}_{true}}\:\:x\left[n\right]\:\:\:\:for\:\:n=\text{0,1},2,\dots\:,N-1$$

For each $\:{\tau}_{true}$, the signal is further subjected to the channel, To simplify the modeling, we consider an AWGN channel, leading to:

28 $$\:{y}_{\tau\:,noised}\left[n\right]={e}^{-i2\pi\:n\tau\:}\:x\left[n\right]+AWGN\:\:\:\:\:for\:\:n=\text{0,1},2,\dots\:,N-1$$

The features for training each model (KSVM, LDA and ANN) are then constructed as follows:

29 $$\begin{aligned}& {Inputs}_{\tau\:}=\left[Re\left({y}_{\tau\:,noised}\left[n\right]\right),{Im}\left({y}_{\tau\:,noised}\left[n\right]\right),\frac{\left|{y}_{\tau\:,noised}\left[n\right]\right|}{\text{max}\left(\left|{y}_{\tau\:,noised}\left[n\right]\right|\right)},\:\frac{\text{arg}\left({y}_{\tau\:,noised}\left[n\right]\right)}{\pi\:}\right]\\& \quad for\:\:n=\text{0,1},2,\dots\:,N-1\end{aligned}$$

These features are essential for accurate and robust machine learning-based CFO estimation, as they effectively capture both amplitude and phase distortions. The real and imaginary components preserve in-phase and quadrature (I/Q) information, crucial for modeling CFO-induced phase shifts in complex-valued OFDM signals. Normalized magnitude ensures learning of relative amplitude variations while making estimation independent of absolute power, enhancing generalization. Normalized phase directly represents CFO-induced rotation, stabilizing learning by keeping values within a consistent range. This feature set enhances precision, noise resilience, and performance across different conditions while supporting various modulation schemes with only minor adjustments to input features. Additionally, it is computationally more efficient than deep learning methods like CNNs.

The target for each input feature set is the corresponding value of τ represented as:

30 $$\:{target}_{\tau\:}=\tau\:$$

The input feature set and corresponding target values are used to train the models, enabling them to predict τ for new inputs, with a table mapping classes to their respective CFO values in KSVM and LDA.

*Additional information can be found in the hyperparameters table within the results section.*

5) **Proposed method (2) Three-step-classifier-based CFO estimator**.

Machine learning-based CFO estimation methods, including single-step KSVM, LDA, and ANN-based estimators, aim to learn the relationship between $\:{Inputs}_{\tau\:}$ and actual CFO. A key insight is that narrowing the CFO search range improves estimation accuracy. This can be demonstrated as follows:

Let the estimated CFO be $\:\widehat{\varDelta f}$ ​, and define the estimation error as:

31 $$\epsilon=\widehat{\varDelta f}-\varDelta f$$

The MSE of CFO estimation is given by:

32 $$\:MSE=E{(\widehat{\varDelta f}-\varDelta f)}^{2}$$

The search range significantly affects MSE; a larger range increases model complexity, requiring more training data and often leading to higher estimation errors due to overfitting. Conversely, narrowing the CFO range allows the model to focus on a smaller subset of values, reducing estimation variance and improving precision. Mathematically, if the search range is reduced from [− F, F] to [− F′,F′] where F′< F, the variance of the estimated CFO decreases:

33 $$\:Var\left(\widehat{\varDelta f}\right)\approx\:\frac{{\sigma}^{2}}{F}$$

where σ2 represents noise and model uncertainty. A smaller range F′ results in a lower variance, thus improving accuracy.

For classification-based estimators (LDA, KSVM), the separability of CFO classes is crucial for accuracy. The probability of misclassification is given by:

34 $$\:{P}_{error}\approx\:Q\left(\frac{{d}_{min}}{\sigma\:}\right)$$

Where $\:{d}_{min}$​ is the minimum distance between CFO classes in feature space, $\:\sigma\:$ is the feature space noise, and Q(⋅) is the Q-function.

When the CFO estimation range is large, the class distances $\:{d}_{min}$​ ​shrink due to overlapping decision boundaries, increasing $\:{P}_{error}$. By decreasing the CFO range, class separation improves, reducing the probability of misclassification.

For ANN-based estimators, the variance of ANN estimation is proportional to the CFO search range F:

35 $$\:{\sigma}_{ANN}^{2}\propto\:\frac{F}{{N}_{training}}$$

where $\:{N}_{training}$​ is the number of training samples. Since MSE is directly related to estimation variance:

36 $$\:MSE\propto\:\frac{F}{{N}_{training}}$$

Thus, reducing F results in a lower MSE, improving estimation accuracy.

**Fig. 3:**
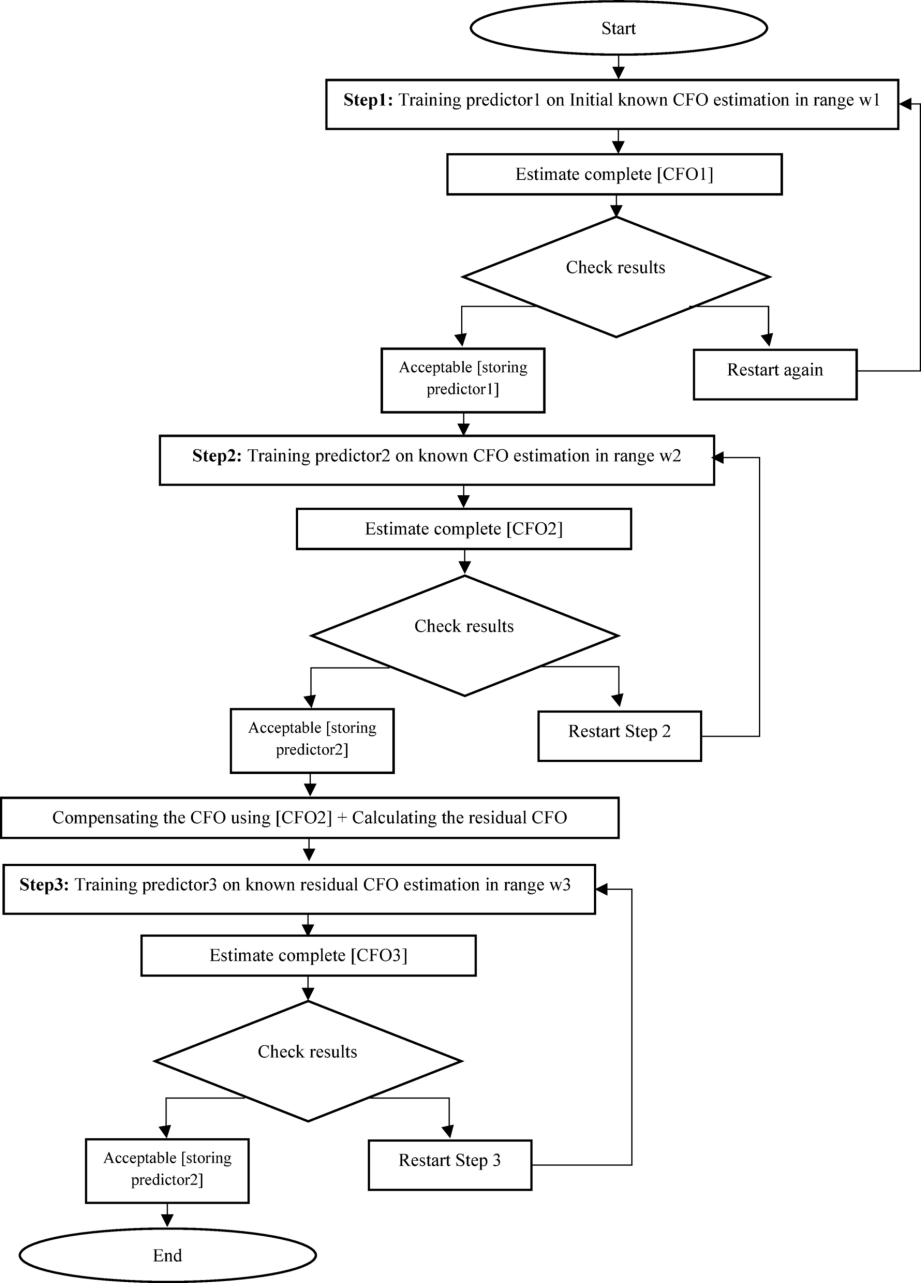
Training procedure of proposed method (2).

**Fig. 4:**
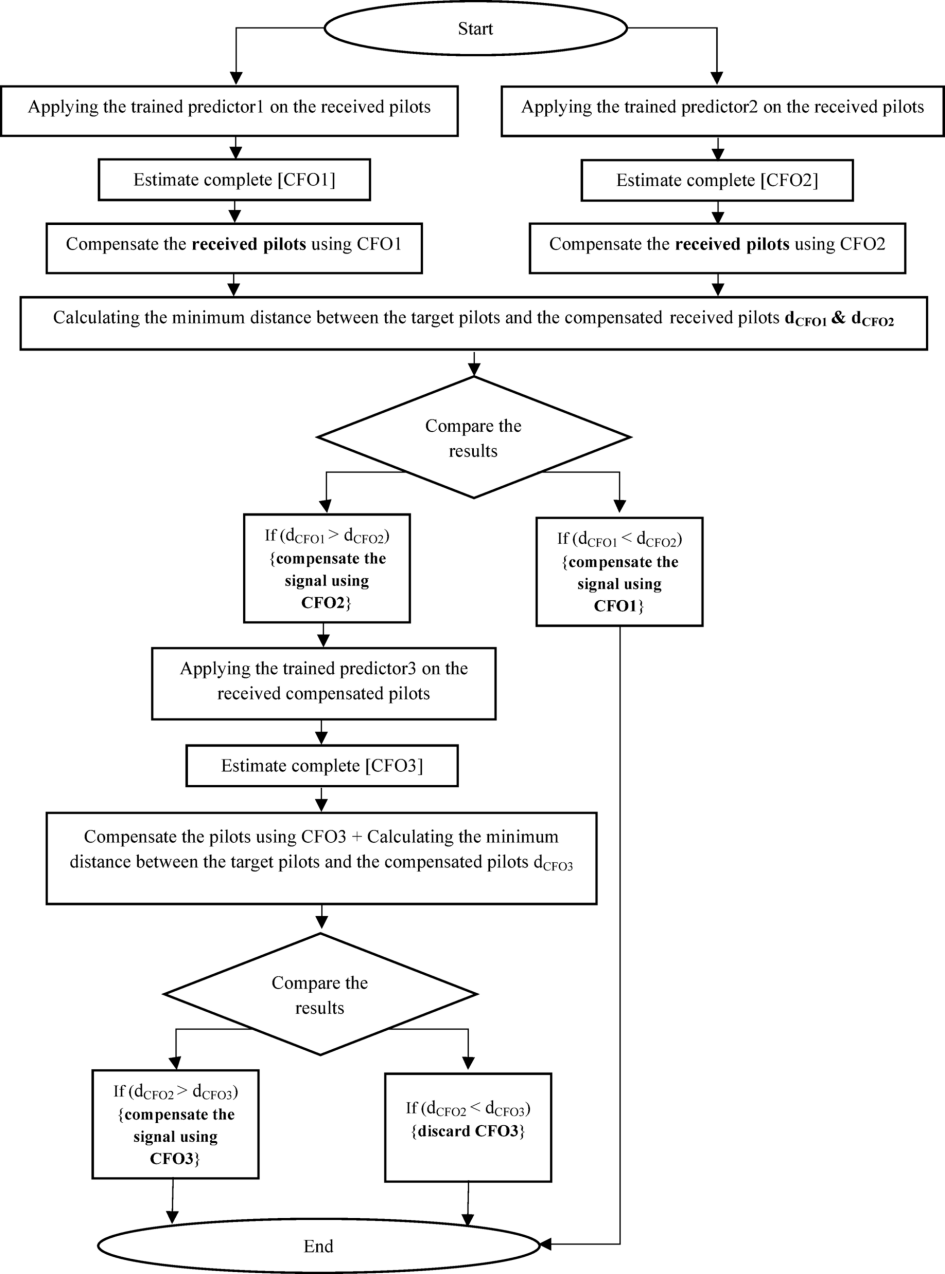
Estimation procedure of proposed method (2).

Leveraging the previous key insight, the proposed method (2) follows a three-step process to achieve optimal outcomes, consisting of one initial step and two subsequent fine steps. Initially, we perform the first step involving larger step sizes. Subsequently, the initial estimation serves as a reference point for refining the estimation within a narrower range by using smaller step sizes to enhance accuracy. The refined estimation from this stage then guides the subsequent step, forming a continuous iterative process. The fine steps can be iterated based on factors such as channel conditions, training efficacy, and estimation precision; however, our approach limits the process to one initial and two fine steps. The iteration may be halted if the estimation significantly deviates from the target pilots in the subsequent step. Our Proposed Method 2 consists of two main phases: a training phase and an estimation phase, each illustrated in separate flowcharts (Figure 3 for training and Figure 4 for CFO estimation). The method employs three predictors: Predictor1, Predictor2, and Predictor3. Predictor1 is used for the initial estimation of the CFO, while Predictor2 and Predictor3 are utilized for fine-tuning the estimation. Depending on the specific implementation, these predictors can be based on KSVM, LDA, or ANN, corresponding to the Three-step-KSVM, Three-step-LDA, or Three-step-ANN CFO estimators, respectively.

During the ***training*** *phase*, received pilots are introduced to a known CFO and the target pilots are identified. A CFO range (w1) is established, and the features and targets (as illustrated before in equations from 26 to 30) are used to train predictor1. A prediction is made based on the known CFO, denoted as CFO1. The residual CFO1 is then calculated, and training continues or ceases based on the accuracy of the estimation. CFO1 is utilized as a reference point to establish the second fine range, denoted as w2 = CFO1 ± x1. For an AWGN channel at 10 dB SNR, the value of x1 is selected to be ± 3 times the residual CFO. Subsequently, predictor2 is trained on the actual features and targets within this updated range, w2.

Following the completion of training, a prediction is made based on the known CFO, which we denote as CFO2. The residual CFO2 is then calculated, and the training process is either resumed or halted based on this residual CFO2. Next, a compensation adjustment is applied to the signal using CFO2. The residual CFO2 acts as a key reference for defining the third fine range, w3 = ± x2. In an AWGN channel with a 10 dB SNR, x2​ is selected as ± 2 times the residual CFO2. Predictor3 is then trained on the real-valued features and targets within this new range, w3. Once training is complete, a prediction is made based on the known residual CFO2, referred to as CFO3. The residual CFO3 is calculated, and the training process is adjusted accordingly based on this value. It is important to note that the values of x1​ and x2​ are not fixed, adjusting based on channel conditions and training performance. In more challenging channel conditions, their values are extended to 4–5 times the residual CFO for enhanced accuracy. These parameters are vital to the three-step-system’s operation—if not carefully chosen, the system defaults to a single-step estimator.

Next, the ***estimation*** *procedure* involves applying the received signal to both Predictor1 and Predictor2 in order to estimate CFO1 and CFO2. Compensation is then applied only to the known pilots before calculating the distances for both CFOs. The corrected pilots using CFO1 and CFO2 ($\:\left[\:\text{a}\text{r}\text{g}\left({y}_{c}\left[n\right]\right)\right]$) are compared with the target pilot ($\:\left[\:\text{a}\text{r}\text{g}\left({y}_{t}\left[n\right]\right)\right]$), and if CFO1 is deemed more accurate, the estimation process is finalized with CFO1. Conversely, if CFO2 is more accurate, compensation using CFO2 is performed on the signal, and the signal is input to Predictor3 for CFO3 prediction. After that, the compensated pilots by CFO2 are compared with the compensated pilots by CFO2 adjusted by CFO3. If the pilots compensated by CFO2 is deemed more accurate, the estimation process is finalized with CFO2. Conversely, if the pilots compensated by CFO2 and CFO3 is more accurate, the frame is compensated with CFO3 and its value included in the final estimated CFO. Subsequently, the final CFO is established and utilized to compensate the incoming frames. To guarantee precise CFO removal, the distance calculation is repeated across multiple frames (as part of a tracking procedure) to confirm that the CFO remains stable and accurate for future frames. If the CFO starts to deviate significantly from the target pilots, the estimation process can be repeated

**More details are presented in the hyperparameters table in the results section.**

## Results and discussion

The implementation is conducted in MATLAB, and this section is organized into two subsections. Subsection A outlines the evaluation method used to test all CFO estimation approaches. Subsection B presents and compares the results of CFO estimation, analyzing the performance of each method under identical conditions.

***A. Evaluation method***.

The evaluation of all methods is conducted using the Monte Carlo Testing Procedure. The details of the testing process used, are as follows:

**Step 1: Monte Carlo Simulation Setup**.

The number of Monte Carlo iterations is defined as:

***M = 10***,***000***.

RMSE accumulators for each CFO estimation method are initialized as:

37 $$\begin{aligned}& \sum{RMSE}_{m}=0\\& m\in\:\{Single\:Step\:ANN,Single\:Step\:LDA,\\&Single\:Step\:KSVM, Three\:Steps\:ANN,Three\:Steps\:LDA,\\&Three\:Steps\:KSVM,AF,CC,CFOest\} \end{aligned}$$

**Step 2: Signal Generation**.

For each iteration i = 1, 2, …, M:

**1. Random CFO Selection**.

A true CFO value $\:{\tau}^{\left(i\right)}$ is randomly chosen from a predefined range:

38 $$\:{\tau}^{\left(i\right)}\:\sim\:P\left(\tau\:\right)$$

where $\:P\left(\tau\:\right)$ represents the probability distribution (discrete uniform distribution) over the selected range.

**2. CFO-Affected Signal Generation**.

The received signal, influenced by CFO, is given by:

39 $$\:{y}_{n}^{\left(i\right)}={e}^{-i2\pi\:n{\tau}^{\left(i\right)}}\:\:{x}_{n}\:\:\:\:for\:\:n=\text{0,1},2,\dots\:,N-1$$

where $\:{x}_{n}$ is the transmitted signal.

Next, $\:{\stackrel{\sim}{y}}_{n}^{\left(i\right)}$ is computed as the received signal, influenced by CFO and the channel effect.

For only AWGN channel, $\:{\stackrel{\sim}{y}}_{n}^{\left(i\right)}$can be presented as:

40 $$\:{\stackrel{\sim}{y}}_{n}^{\left(i\right)}={y}_{n}^{\left(i\right)}+{w}_{n}\:\:\:\:\:\:\:\:\:\:\:{w}_{n}\sim\mathcal{C}\mathcal{N}(0,{\sigma}^{2})$$

For Rayleigh fading channel, $\:{\stackrel{\sim}{y}}_{n}^{\left(i\right)}$can be presented as:

41 $$\:{\stackrel{\sim}{y}}_{n}^{\left(i\right)}=\sum_{k=0}^{L-1}h\left[k\right]\:.\:{y}_{n}^{\left(i\right)}\left[n-k\right]+{w}_{n}$$

where $\:h\left[k\right]$ is the discrete-time representation of the Rayleigh fading channel impulse response, and L is the channel length.

**3. Feature Extraction**.

The input feature vector, denoted as $\:{\varvec{f}}^{\left(\varvec{i}\right)}$, is used to construct the input.

**Step 3: CFO Estimation**.

CFO estimation is then conducted for each model m, resulting in the predicted CFO as:

42 $$\:{\widehat{\tau\:}}_{m}^{\left(i\right)}={f}_{m}\left({\varvec{f}}^{\left(\varvec{i}\right)}\right)$$

Where $\:{f}_{m}$ represents the prediction (KSVM, LDA, and ANN) or calculation (CFOest, AF, and CC) function of the respective method.

**Step 4: RMSE Calculation**.

1. **RMSE Accumulation**.

For each method m, the RMSE accumulation is given by:

43 $$\:\sum\:{RMSE}_{m}=\sum_{i=1}^{M}{({\tau}^{\left(i\right)}-{\widehat{\tau\:}}_{m}^{\left(i\right)})}^{2}$$

**2. Final RMSE Computation**.

After all iterations, the final RMSE for each method is computed as:

44 $$\:\sum\:{RMSE}_{m}=\sqrt{\frac{1}{M}}\sum_{i=1}^{M}{({\tau}^{\left(i\right)}-{\widehat{\tau\:}}_{m}^{\left(i\right)})}^{2}$$

Table 2 illustrates all the hyperparameters used in this study.

**Table 2:** The hyperparameters table.

Name	Type	Hidden Layers and Activation Functions	Features and Classes	Back-propagation and training	Additional Notes
ANN	MLP for Classification (Predictor1 = ANN1 Predictor2 = ANN2 Predictor3 = ANN3)	3 Hidden Layers. Number of neurons in each layer $\:{H}_{l}=\lfloor\frac{n+1}{{2}^{l-1}}+0.5\rfloor\:\:\:,l\ge\:1$ Where n is half of the preamble length, $\:l$ represents the hidden layer index, and ⌊⋅⌋ represents rounding to the nearest integer. (e.g., with three hidden layers and 64 pilots, the neuron distribution is: 33, 17, and 9 neurons, respectively) Activation Functions = Hyperbolic Tangent Sigmoid (Hidden), Linear (Output)	**Features**: For ANN1, ANN2 and ANN3, the features for training each **model** are given by: $\:{Inputs}_{\tau\:}=Re\left({y}_{\tau\:,noised}\left[n\right]\right),{Im}\left({y}_{\tau\:,noised}\left[n\right]\right),\dots\:.$ $\:,\frac{\left|{y}_{\tau\:,noised}\left[n\right]\right|}{\text{max}\left(\left|{y}_{\tau\:,noised}\left[n\right]\right|\right)},\:\frac{\text{arg}\left({y}_{\tau\:,noised}\left[n\right]\right)}{\pi\:}\:$ $\:for\:\:n=\text{0,1},2,\dots\:,N-1$ **Classes**: 100 Class in w1 (CFO = 1/700:1/400). 100 Class in w2 (w2 = CFO1 ± x1, x1 = ± 3 times the residual CFO). 100 Class in w3 (w3 = ± x2, x2 = ± 2 times the residual CFO2). In Rayleigh fading channels, x1​ and x2​ are extended to 4–5 times the residual CFO.	Levenberg-Marquardt with Bayesian regularization Trained on 1:25 frames (depending on the channel), with each frame containing 64 pilots.	–
LDA	LDA Classification (Predictor1 = LDA1 Predictor2 = LDA2 Predictor3 = LDA3)	N/A	**Features**: For LDA1, LDA2 and LDA3, the features for training each **model** are given by: $\:{Inputs}_{\tau\:}=Re\left({y}_{\tau\:,noised}\left[n\right]\right),{Im}\left({y}_{\tau\:,noised}\left[n\right]\right),\dots\:.$ $\:,\frac{\left|{y}_{\tau\:,noised}\left[n\right]\right|}{\text{max}\left(\left|{y}_{\tau\:,noised}\left[n\right]\right|\right)},\:\frac{\text{arg}\left({y}_{\tau\:,noised}\left[n\right]\right)}{\pi\:}\:$ $\:for\:\:n=\text{0,1},2,\dots\:,N-1$ **Classes**: 100 Class in w1 (CFO = 1/700:1/400). 100 Class in w2 (w2 = CFO1 ± x1, x1 = ± 3 times the residual CFO). 100 Class in w3 (w3 = ± x2, x2 = ± 2 times the residual CFO2). In Rayleigh fading channels, x1​ and x2​ are extended to 4–5 times the residual CFO. A lookup table is used to set the targets to a class sets, then this table is saved, and used when the model is applied.	Trained on 1:25 frames (depending on the channel), with each frame containing 64 pilots.	Pseudo-inverse for covariance matrix inversion.
KSVM	Kernel Support Vector Machine Classification (Predictor1 = KSVM1 Predictor2 = KSVM2 Predictor3 = KSVM3)	N/A	**Features**: For KSVM1, KSVM2 and KSVM3, the features for training each **model** are given by: $\:{Inputs}_{\tau\:}=Re\left({y}_{\tau\:,noised}\left[n\right]\right),{Im}\left({y}_{\tau\:,noised}\left[n\right]\right),\dots\:.$ $\:,\frac{\left|{y}_{\tau\:,noised}\left[n\right]\right|}{\text{max}\left(\left|{y}_{\tau\:,noised}\left[n\right]\right|\right)},\:\frac{\text{arg}\left({y}_{\tau\:,noised}\left[n\right]\right)}{\pi\:}\:$ $\:for\:\:n=\text{0,1},2,\dots\:,N-1$ **Classes**: 100 Class in w1 (CFO = 1/700:1/400). 100 Class in w2 (w2 = CFO1 ± x1, x1 = ± 3 times the residual CFO). 100 Class in w3 (w3 = ± x2, x2 = ± 2 times the residual CFO2). In Rayleigh fading channels, x1​ and x2​ are extended to 4–5 times the residual CFO. A lookup table is used to set the targets to a class sets, then this table is saved, and used when the model is applied.	Trained on 1:25 frames (depending on the channel), with each frame containing 64 pilots.	RBF kernel.

B. ***Carrier frequency offset estimation result***.

**Fig. 5:**
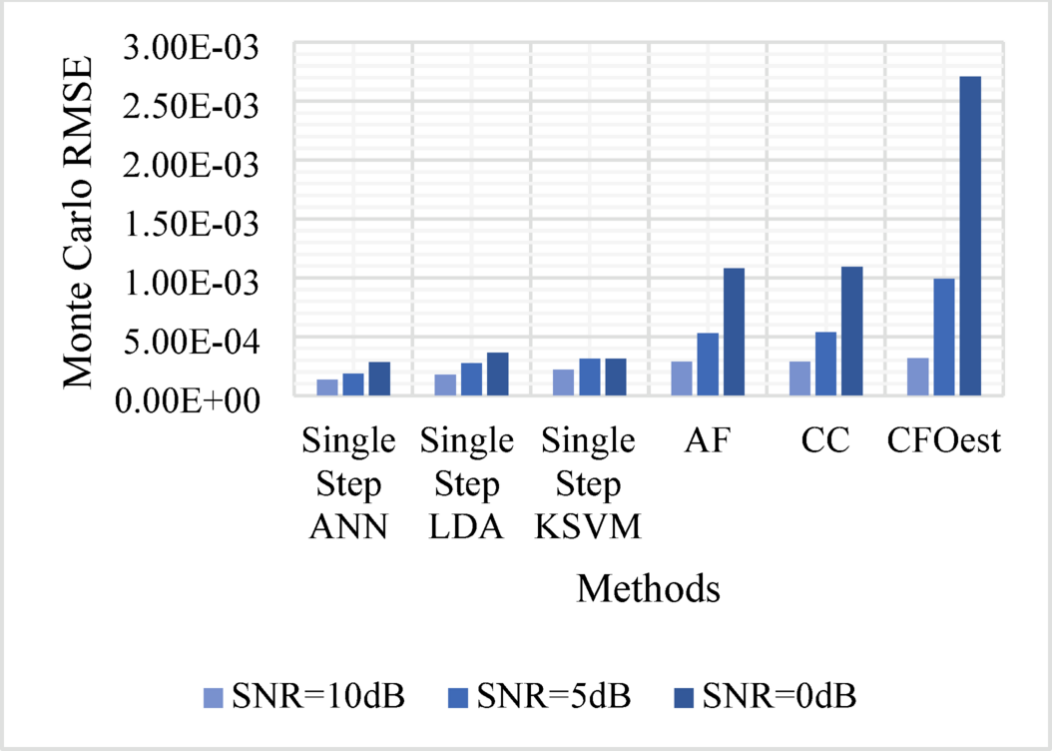
Comparison of Monte Carlo RMSEs in an AWGN channel (Method 1 vs. Traditional Techniques).

**Fig. 6:**
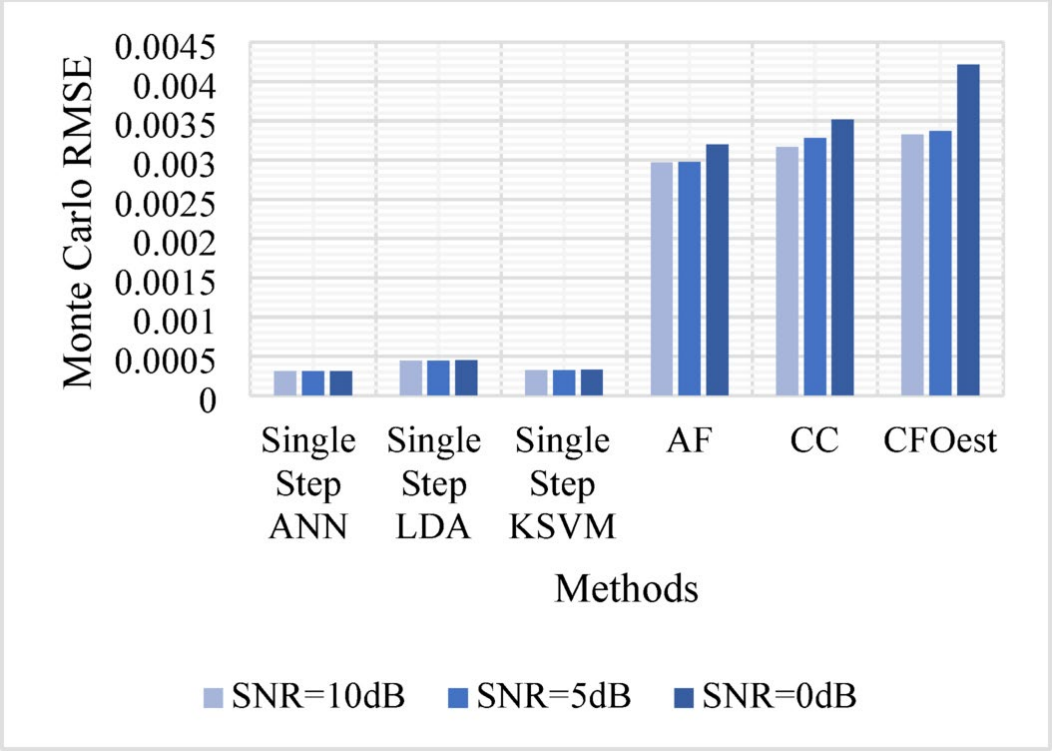
Comparison of Monte Carlo RMSEs in a Flat Rayleigh Fading Channel (Method 1 vs. Traditional Techniques).

**Fig. 7:**
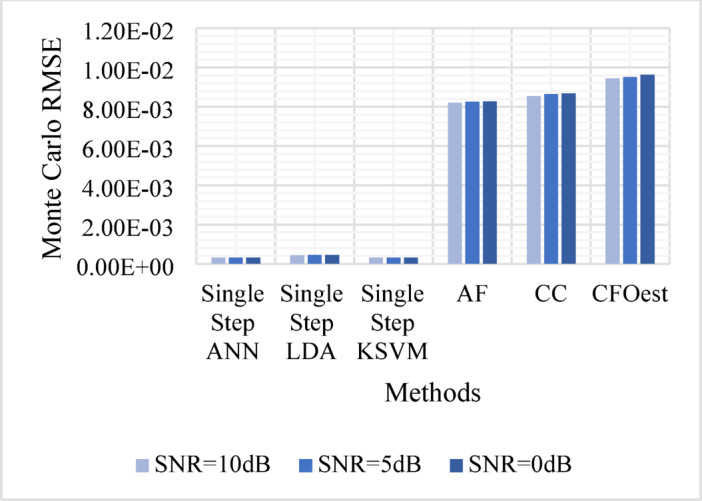
Comparison of Monte Carlo RMSEs in a Frequency-Selective Rayleigh Fading Channel 1 (Method 1 vs. Traditional Techniques).

**Fig. 8:**
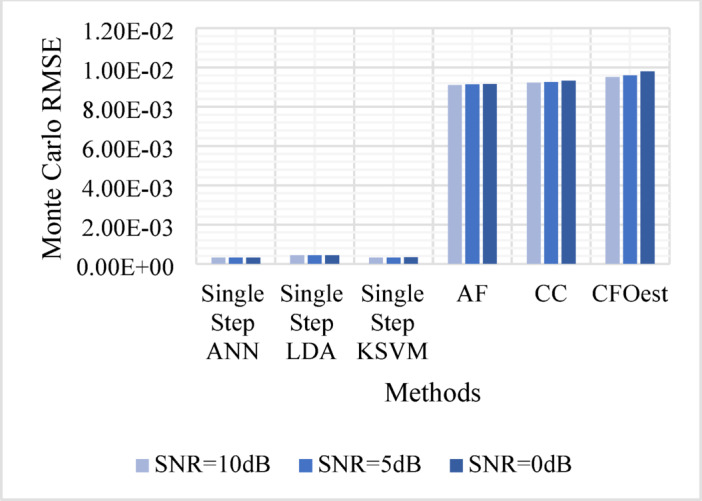
Comparison of Monte Carlo RMSEs in a Frequency-Selective Rayleigh Fading Channel 2 (Method 1 vs. Traditional Techniques).

**Fig. 9:**
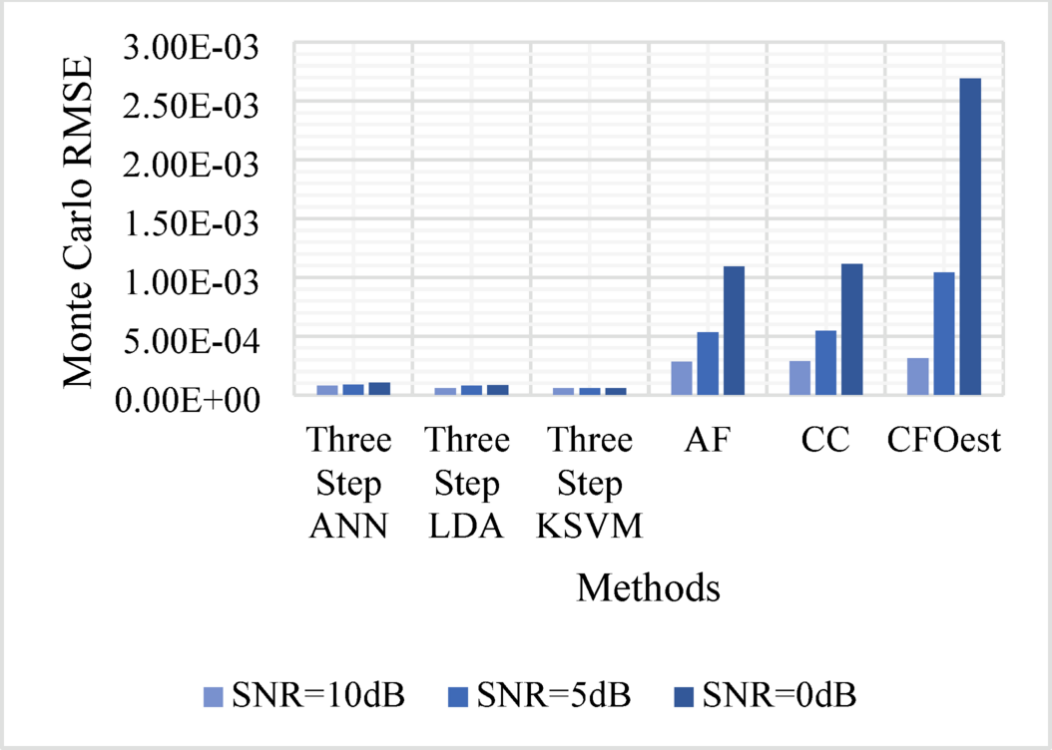
Comparison of Monte Carlo RMSEs in an AWGN channel (Method 2 vs. Traditional Techniques).

**Fig. 10:**
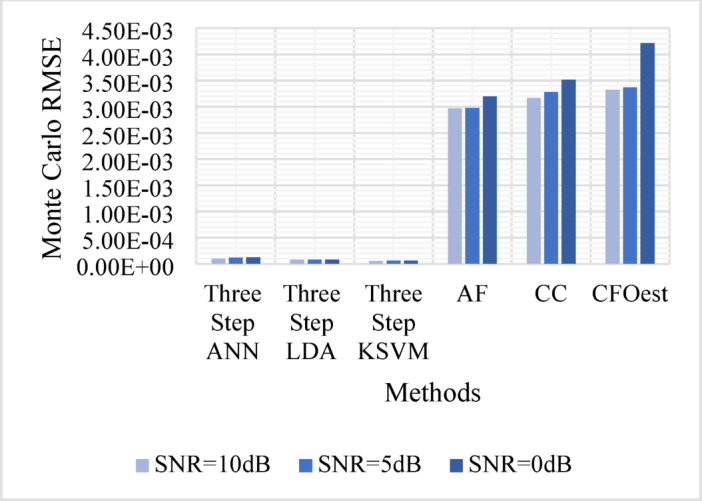
Comparison of Monte Carlo RMSEs in a Flat Rayleigh Fading Channel (Method 2 vs. Traditional Techniques).

**Fig. 11:**
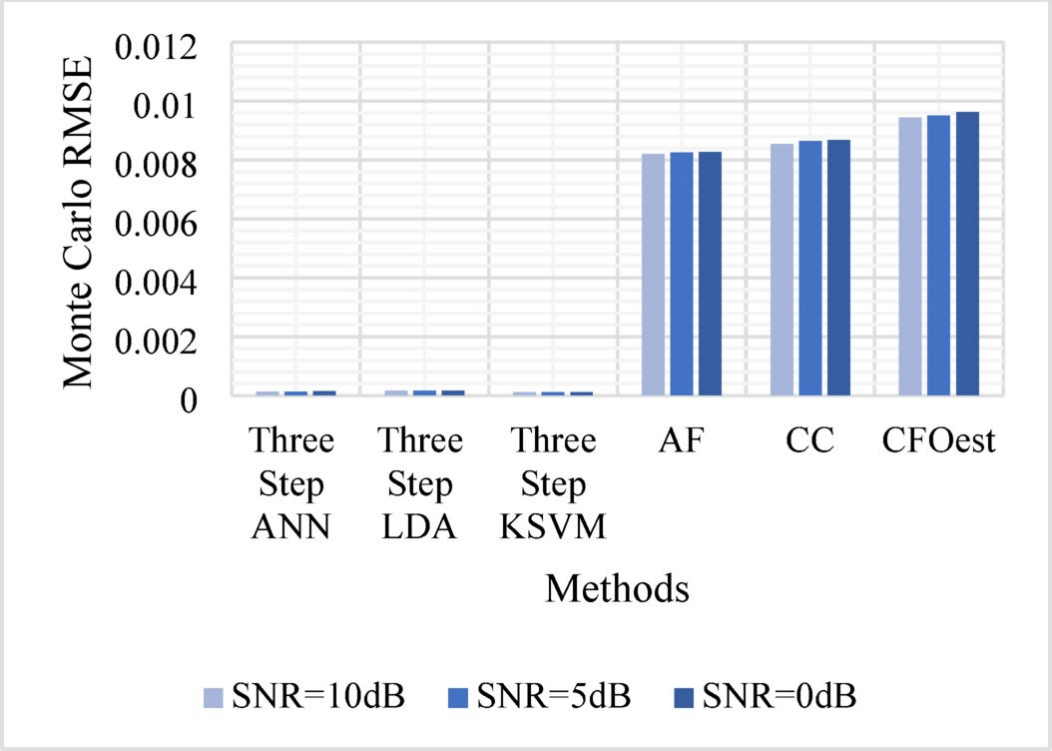
Comparison of Monte Carlo RMSEs in a Frequency-Selective Rayleigh Fading Channel 1 (Method 2 vs. Traditional Techniques).

**Fig. 12:**
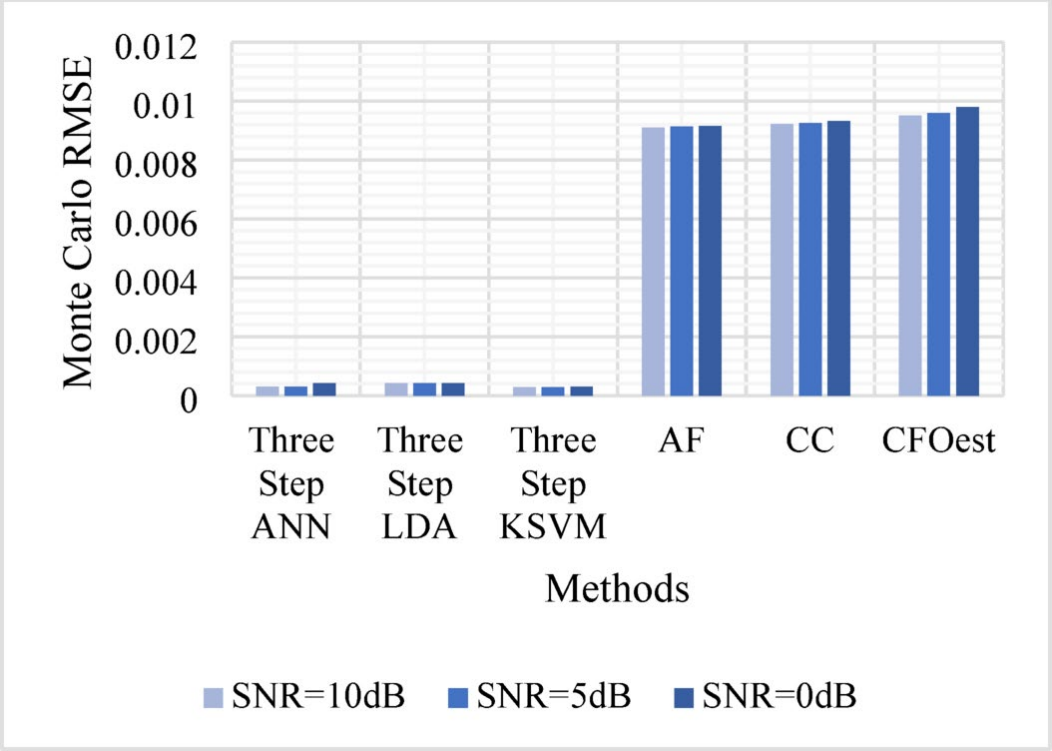
Comparison of Monte Carlo RMSEs in a Frequency-Selective Rayleigh Fading Channel 2 (Method 2 vs. Traditional Techniques).

The Monte Carlo testing results are illustrated in eight figures, comparing the proposed methods with conventional techniques across four different channel conditions. These comparisons are divided into two sets:

1. **First Set** (Figs. 5, 6, 7, and 8).

- Evaluates Method 1 (Single-Step ANN, KSVM, and LDA) against traditional approaches (AF, CC, and CFOest) under various channel conditions, including AWGN, Flat Rayleigh Fading, and Frequency-Selective Rayleigh Fading (Channels 1 and 2). The characteristics of each Rayleigh fading channel are detailed in Table 3.

2. **Second Set** (Figs. 9, 10, 11, and 12).

- Assesses **Method 2** (Three-Step ANN, Three-Step KSVM, and Three-Step LDA) under the same conditions, comparing its performance to the same traditional techniques.

**Performance analysis is illustrated as follows**:

- Figure 5: Shows the Monte Carlo RMSE performance of **Method 1** under AWGN at 10 dB, 5 dB, and 0 dB. The results indicate significantly lower RMSE across all SNR levels compared to traditional methods, highlighting improved robustness to noise. In contrast, AF, CC, and CFOest exhibit high RMSE values, with CFOest suffering the most severe degradation at 0 dB.
- Figures 6, 7 and 8: Present the RMSE performance of **Method 1** under Flat Rayleigh Fading and Frequency-Selective Rayleigh Fading Channels 1 and 2, respectively. The machine learning models trained on these channels perform better in the Flat Rayleigh Fading scenario. Conversely, AF, CC, and CFOest exhibit high RMSE values across all cases, with even worse performance in Frequency-Selective Rayleigh Fading Channels 1 and 2, particularly for CFOest.
- Figure 9: Demonstrates the RMSE performance of **Method 2** under AWGN at 10 dB, 5 dB, and 0 dB. The results show that **Method 2** outperforms both **Method 1** and the traditional techniques, achieving significantly lower RMSE across all SNR levels.
- Figures 10, 11 and 12: Show the RMSE performance of **Method 2** under Flat Rayleigh Fading, and Frequency-Selective Rayleigh Fading Channels 1 and 2, respectively. Similar to **Method 1**, the machine learning models achieve better results in the Flat Rayleigh Fading scenario. Traditional methods continue to exhibit high RMSE values, with CFOest performing the worst in Frequency-Selective Rayleigh Fading Channels 1 and 2.

**Table 3:** Simulation parameters for flat and Frequency-Selective Rayleigh fading channels.

Parameters (Normalized System)	Flat Rayleigh Fading Channel	Frequency-Selective Rayleigh Fading Channel 1	Frequency-Selective Rayleigh Fading Channel 2
Doppler Shift (× Rs) Hz	0.1	1	3
Number of Multipath Components	1	5	8
Path Delays (sec)	[0]	[0,0.3Ts,0.7Ts,1.5Ts,2Ts]	[0,0.2Ts,0.5Ts,0.9Ts,1.3Ts,1.8Ts,2.5Ts,3Ts]
Average path gains (dB)	[-3]	[0,−3,−6,−9,−12]	[0,−4,−8,−12,−16,−20,−25,−30]

**Fig. 13:**
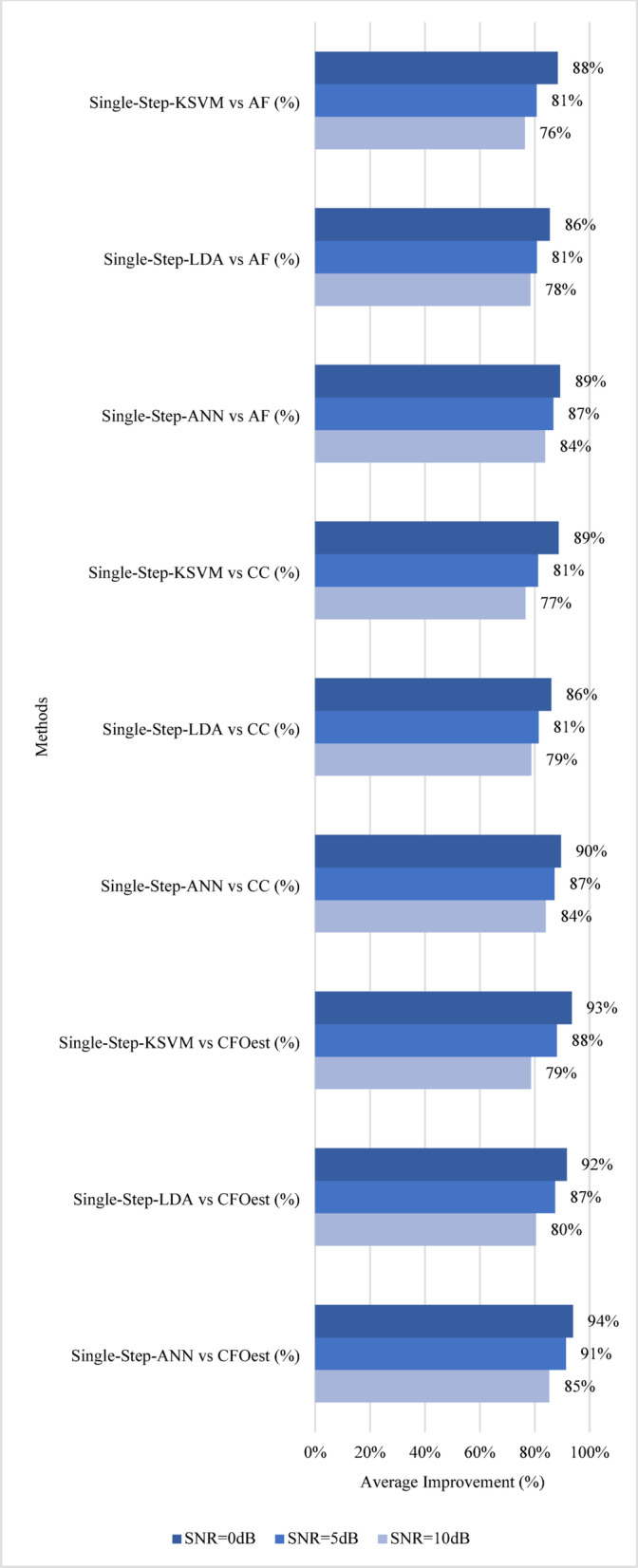
Impact of Single-Step Methods on Estimation Accuracy.

**Fig. 14:**
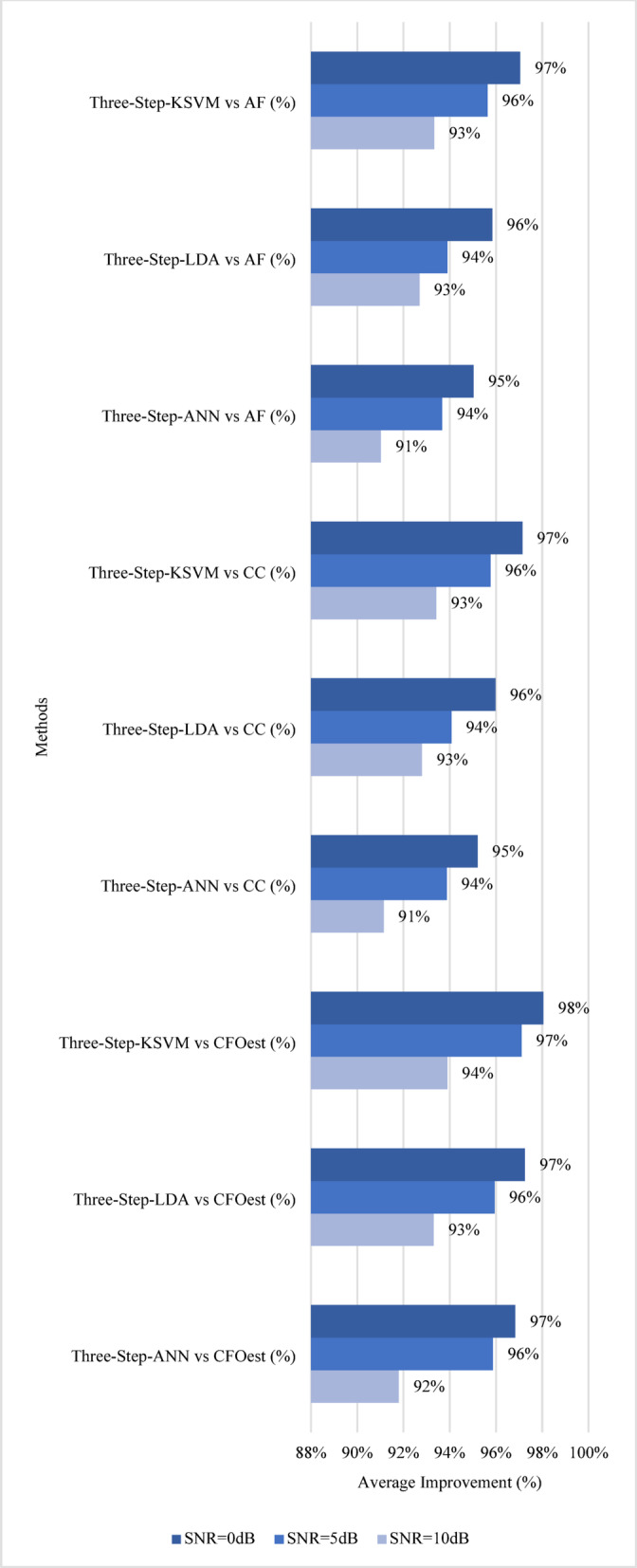
Impact of Three-Step Methods on Estimation Accuracy.

**Fig. 15:**
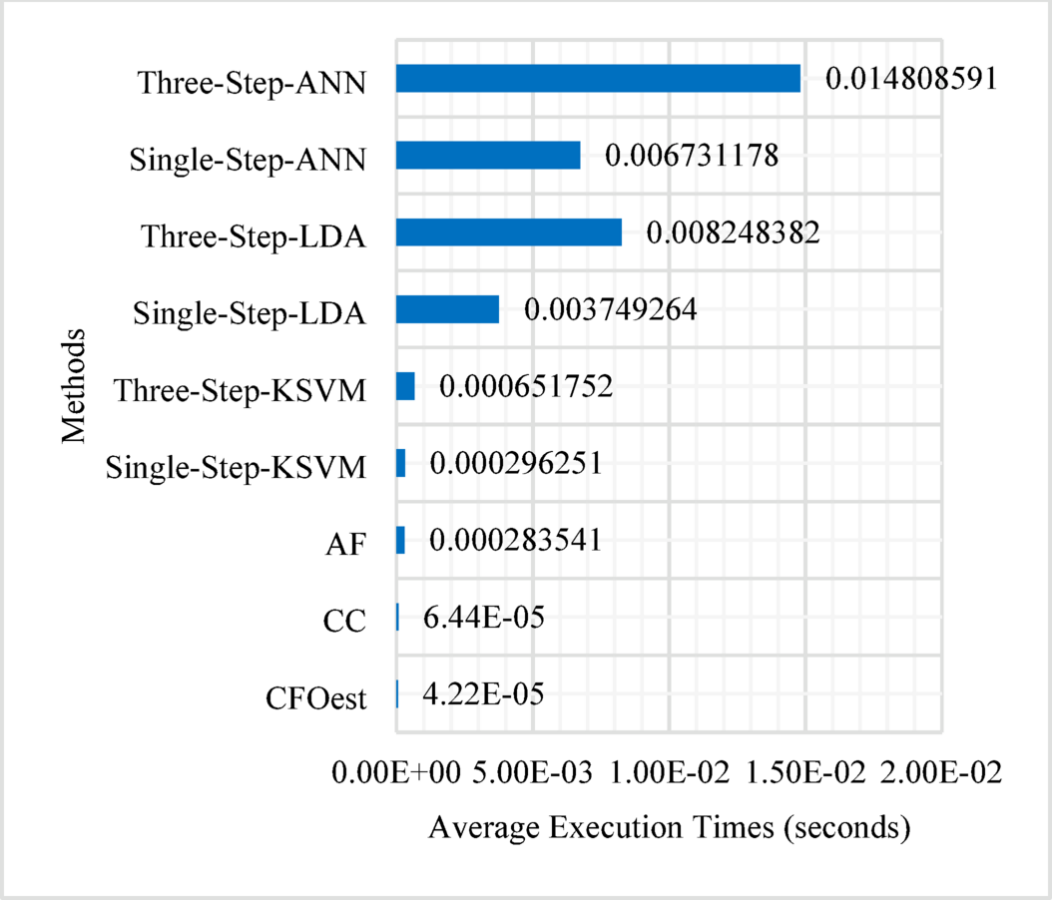
Average execution times excluding time spent training the models.

**Fig. 16:**
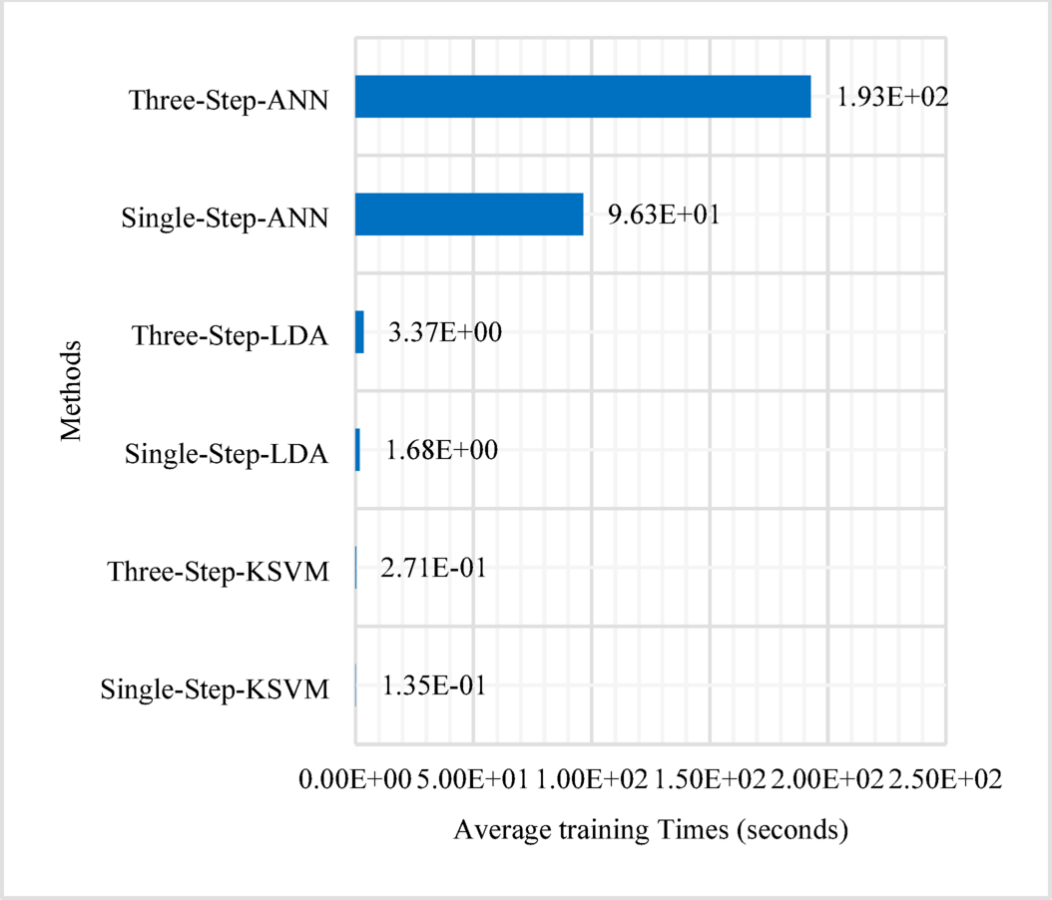
Time spent training the models.

From the previous eight figures we can conclude that, **Method 2** demonstrates superior performance compared to **Method 1** and significantly outperforms conventional techniques across all evaluated conditions.

For a more comprehensive analysis, the following discussion evaluates both **estimation accuracy** and **robustness**. Figures 13 and 14 illustrate the results, highlighting the **average percentage improvement** over conventional methods in terms of estimation accuracy. For Method 1 (Single-Step), Single-Step-ANN achieves the highest average accuracy improvement across all SNR levels in all channels, peaking at 94% when SNR = 0 dB, followed closely by Single-Step-KSVM at 93% and Single-Step-LDA at 92%. When compared to CC, Single-Step-ANN again leads with a 90% improvement, while Single-Step-KSVM and Single-Step-LDA achieve 89% and 86%, respectively. Against AF, Single-Step-ANN remains the best performer at 89%, followed by Single-Step-KSVM at 88% and Single-Step-LDA at 86%. For Method 2 (Three-Step), Three-Step-KSVM consistently achieves the highest average accuracy improvement, reaching 98% at SNR = 0 dB, while Three-Step-ANN and Three-Step-LDA follow closely at 97%. When compared to CC, Three-Step-KSVM again leads with a 97% improvement, Three-Step-LDA follows at 96%, and Three-Step-ANN reaches 95%. Against AF, Three-Step-KSVM maintains the highest average accuracy improvement at 97%, while Three-Step-ANN and Three-Step-LDA achieve 95% and 96%, respectively.

In terms of **robustness**, classifier-based methods demonstrate significantly greater resistance to SNR and channel degradation compared to conventional methods. For Method 1, Single-Step-ANN and LDA maintain a stable RMSE increase of approximately 28%, while Single-Step-KSVM exhibits even better resilience with only a 12.16% increase. In contrast, AF, CC, and CFOest suffer extreme performance degradation, with RMSE increases exceeding 278%, 281%, and 759%, respectively. For Method 2, the results closely resemble those of Method 1. Three-Step-KSVM demonstrates the highest robustness, maintaining stable performance across different SNR and channel conditions. In contrast, AF, CC, and CFOest suffer severe performance deterioration, highlighting their extreme vulnerability to SNR and channel degradation.

Figure 15 illustrates the average execution times for CFO estimation, with training time excluded (Inference Latency), whereas Fig. 16 highlights the time required for model training (Training Latency). All time measurements were performed on a PC equipped with an AMD Ryzen 5 5600G processor (3.90 GHz) and 16GB of RAM. The execution time is determined based on 10,000 estimation processes, with the average taken. The figures indicate that the CFOest, CC, AF, and KSVM-based CFO estimators exhibit relatively low execution times. In contrast, the LDA-based and ANN-based CFO estimators require significantly more time, with the ANN-based estimators demonstrating the longest execution duration among all methods.

It is important to note that Practical deployment requires balancing accuracy, computational cost, and processing time, as different applications have varying priorities. High-precision systems demand highly accurate but computationally intensive estimators, while resource-constrained environments benefit from simpler, lower-power alternatives. The choice of an estimator should align with system constraints, considering factors like hardware limitations and energy efficiency. Additionally, the three-step estimator models could also be hybrid, further enhancing their effectiveness in real-time applications.

## Conclusion

This study evaluated both traditional and machine learning-based CFO estimation techniques, demonstrating that machine learning significantly enhances accuracy, particularly in challenging channel conditions. ANN-based estimators excel in broader estimation ranges, while KSVM-based estimators provide superior fine estimation with lower computational complexity. LDA-based estimators perform well in high-SNR and favorable channel conditions but are outperformed in more challenging channel conditions.

The introduction of a three-step machine learning framework significantly enhanced accuracy, outperforming both traditional methods and single-step models. Simulations validated its effectiveness, emphasizing the ANN-based estimator as the optimal choice for initial estimation due to its superior accuracy over wider CFO ranges, while KSVM demonstrated robust and stable overall performance. Among KSVM-based, LDA-based, and ANN-based estimators, KSVM-based methods achieved the best performance in narrow CFO ranges while maintaining the lowest latency. In contrast, traditional methods exhibited the highest RMSE, particularly in low-SNR environments and challenging channel conditions, making them the least reliable option. Future research could explore hybrid machine learning frameworks to balance accuracy, complexity, and adaptability, as well as investigate the real-time implementation of the three-step framework.

## Data Availability

Available upon reasonable request from the corresponding author.
